# TAKING THE HIGH (SCHOOL) ROAD: DEVELOPING INTERGENERATIONAL INTEREST FOR CAREERS IN AGING

**DOI:** 10.1093/geroni/igz038.2100

**Published:** 2019-11-08

**Authors:** Rona J Karasik, Phyllis A Greenberg

**Affiliations:** Saint Cloud State University, Saint Cloud, Minnesota, United States

## Abstract

Invisibility and ageism are ever-present challenges to developing a work-force commensurate with the expanding older population (Augustin & Freshman, 2016). Intergenerational and experiential endeavors (e.g., Simulations, Service-Learning, Careers in Aging Week) can counter stereotypes while highlighting gerontology career opportunities. Focusing workforce development efforts on the oft-overlooked population of high school students (Zhang, 2015) has numerous advantages, including: (1) identifying aging-related career paths prior to college; (2) reaching students eligible to become CNAs/PCAs; (3) uncovering potential benefits (e.g., scholarships, tuition reimbursements) agencies may offer to student-employees and (4) countering aging stereotypes. This presentation reports on a multi-faceted undertaking which includes collaborating with local service agencies on a regional aging-workforce initiative, bringing high-school students to a college campus for CIAW events, participating in local/regional career events focused on high school students, and developing/implementing experientially-rich learning opportunities such as a college-credit bearing introductory course on aging to be taught at the high school.

